# *Dirofilaria repens* Parasite: Review with Emphasis on Ultrasound Findings with Looking for Worm Mobility

**DOI:** 10.5334/jbsr.4072

**Published:** 2026-03-26

**Authors:** Jacques Malghem, Bruno Vande Berg, Benoît Lengelé, Frédéric Lecouvet

**Affiliations:** 1 Department of Medical Imaging, Cliniques Universitaires Saint Luc, UCLouvain, Brussels, Belgium; 2 Department of Radiology, CHC Mont Legia, Liège, Belgium; 3 Department of Plastic, Reconstructive and Aesthetic Surgery, Cliniques Universitaires Saint Luc, UCLouvain, Brussels, Belgium

**Keywords:** Dirofilaria, parasite, human dirofilariasis, soft tissues tumor, imaging, ultrasound

## Abstract

Human dirofilariasis is caused by infection with a parasite relatively common in dogs. Occasional transmission of larvae to humans occurs via an insect bite and development of an immature worm at the bite site. The disease, once confined to southern Europe, is becoming increasingly widespread, particularly due to global warming. Clinical signs are nonspecific and laboratory findings usually normal. Imaging shows pseudotumor features also nonspecific on computed tomography (CT) and magnetic resonance imaging (MRI) scans. Ultrasound may suggest the diagnosis by the presence of double echogenic ‘rail’ images. However, only spontaneous motility of the parasite observation can confirm the diagnosis.

## Introduction

Human infections by *Dirofilaria repens* parasites are relatively rare, but they are on the rise in Europe. Their detection in medical imaging can be crucial.

We present a review of all the characteristics of the parasite and the human disease it transmits, with morphological, epidemiological, clinical, and diagnostic aspects, including imaging methods. We will emphasize the ultrasound aspect and the possible in vivo demonstration of the parasite’s mobility in case of superficial human localization, with the presentation of an exemplary clinical case.

## 1. Animal and Human Disease

Dirofilariasis is a parasitic disease involving wild or domestic carnivorous animals, particularly dogs (the main reservoir) and, more rarely, cats. Human contamination is rather anecdotal [1, 2].

The transmission of the parasite occurs through mosquitoes, with different species according to the region (especially *Aedes*, *Culex*, and *Anopheles*).

Parasite larvae (microfilariae) present in the blood of the infected animal are sucked in by a mosquito, which transmits them through a bite to a new host [1, 3, 4].

There are several dozen varieties of *Dirofilaria*, but the most important in terms of pathogenicity are the immitis and repens varieties, which have different target organs.

For the *Dirofilaria immitis* species, the final target organs are mainly the lungs and heart. It is a serious disease for dogs, which can be fatal. In humans, this parasite mainly forms pulmonary nodules, which are usually asymptomatic and can mimic cancer on X-rays. The rare, occasional symptoms are variable (cough, hemoptysis, fever, or various discomforts) [2].

For the species *D. repens*, the human occurrence of which is more frequent than that of *D. immitis*, the target organ is the hypodermis [1, 2, 5]. Lesions develop in the hypodermic tissue anywhere near the bite site. The most common site is the head region (nearly half of the cases), particularly the ocular region, which represents about one-third [1, 2, 4, 6, 7].

Humans constitute a dead-end for the parasite because the parasite rarely reaches sexual maturity and dies after 2 to 8 years. If the parasite reaches maturity, it is generally not fertilized. Indeed, for reproduction to occur, mosquito bites must release enough larvae that successfully reach maturity, and parasites can meet and mate. Therefore, microfilaremia is exceptional [4, 5, 7, 8].

## 2. Epidemiology

*D. immitis* predominates in America, especially in the United States, as well as in Japan.

In Europe, cases of *D. repens* overwhelmingly dominate. Before the year 2000, most cases were observed in Mediterranean regions. In subsequent years, Mediterranean cases increased further, but other cases also appeared in Central and Eastern Europe, and the incidence is currently extending toward Northern Europe (northern France, Germany, England, Baltic countries, and also appears in Asian countries) [2–4, 6, 7, 9, 10, 11].

The transmission of *Dirofilaria* depends on the presence of (1) a minimum number of infected dogs containing adult worms producing microfilariae and (2) mosquitoes capable of transmitting them. An increase in the incidence of cases could result from sociological factors, including the number of dogs and the increase in their movement, facilitated by current European regulations on travel with pets [2, 12].

The other important factor is the increase in insect vectors, which could be linked in particular to bans on insecticides or, conversely, to the development of resistance in the event of overuse. A particular human activity, the intercontinental transport of used tires containing mosquito nests, introduced into Europe a few years ago an Asian mosquito (*Aedes albopictus*, known as the ‘tiger mosquito’), which is thought to play a very important role in the increasing transmission of *Dirofilaria* in Europe [13].

**Figure 1:**
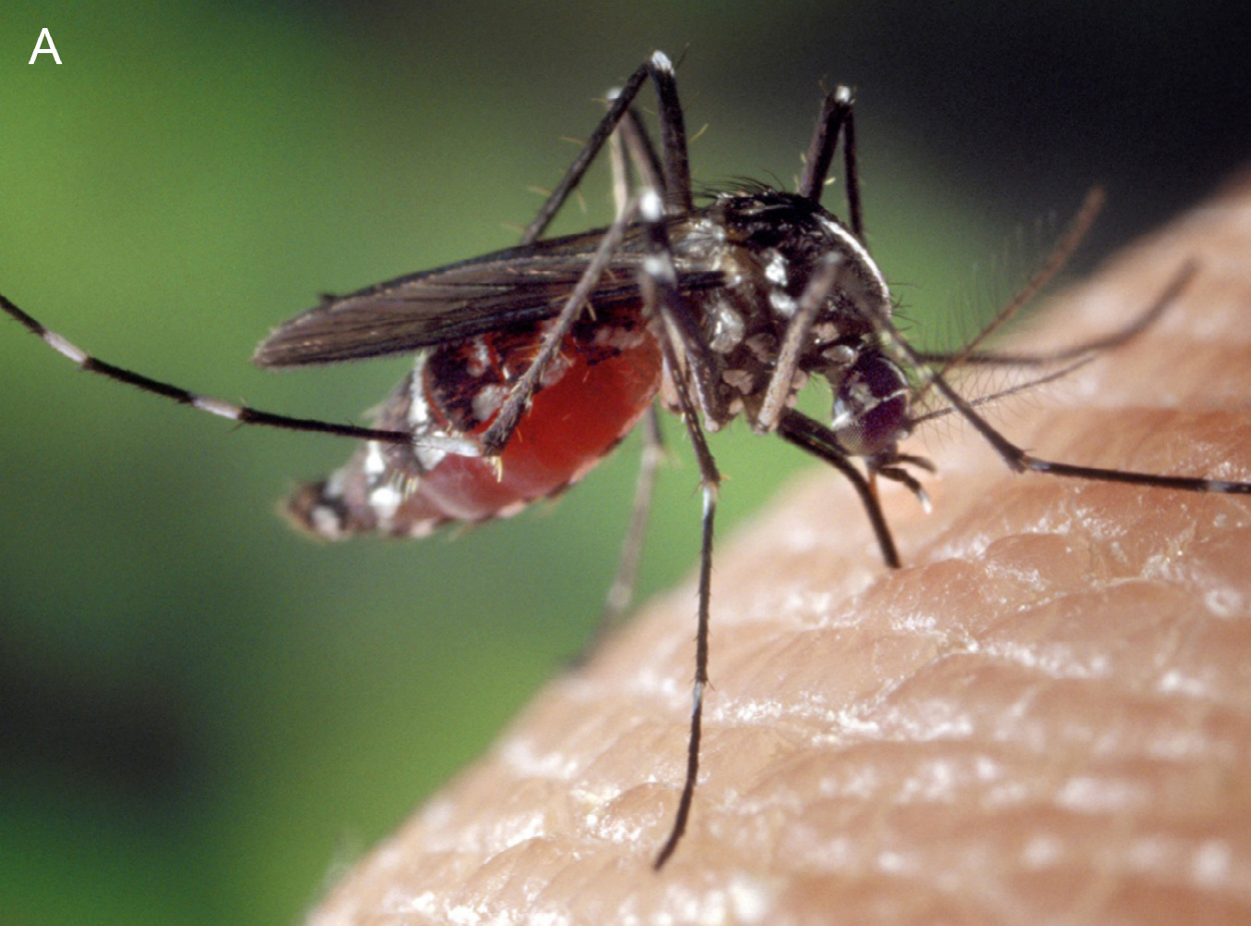

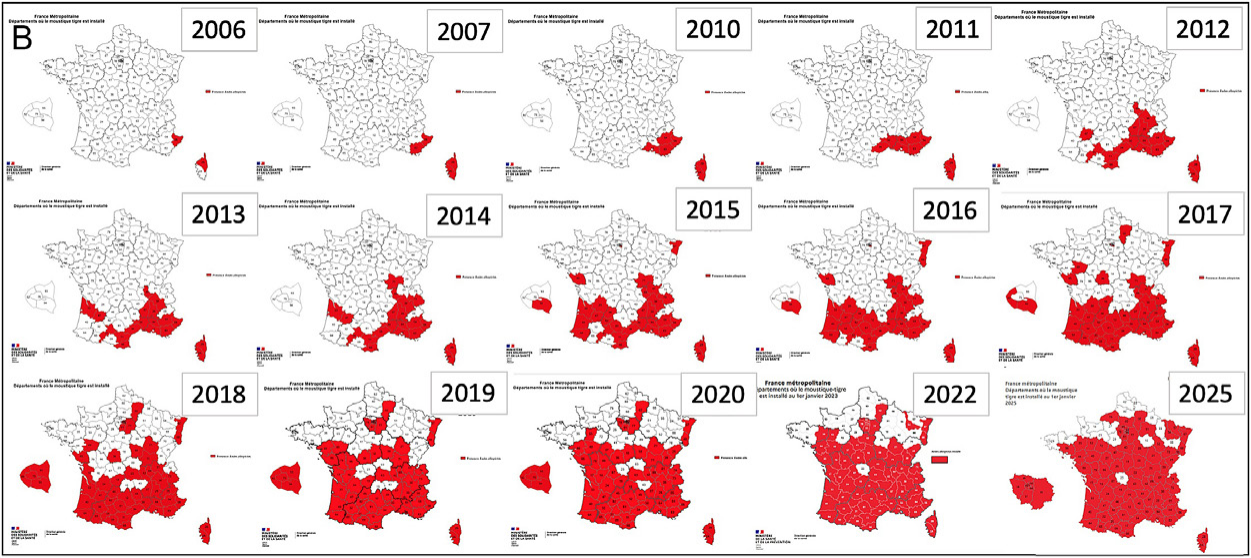
Tiger mosquito. **(A)** Its appearance. **(B)** Illustration of its distribution in France in recent years, from 2006 to 2025, showing its almost exponential expansion from south to north.

Overall, the increase in the prevalence of *Dirofilaria* infections appears to be particularly linked to climate change, as the development of these insects requires a temperature of 14°C [2, 10, 14, 15]. This increasing risk is well illustrated by the geographical extension in France of the infestation by tiger mosquitoes, which has experienced an almost exponential expansion (Figure 1) [16].

## 3. Clinic, Biology, Identification, and Therapeutics

The parasite, usually a single one, develops within a few weeks. Clinical manifestations depend on the location of the worm in the organs or tissues.

In the case of ocular dirofilariasis, symptoms are recurrent ocular redness, pain, discomfort, itching, and foreign body sensation. The mobility of the parasite can be observed directly [17, 18].

In the case of superficial localization in soft tissues, after an initial inflammatory tissue reaction, sometimes itchy or accompanied by burning sensations, a hypodermal nodule of 1–3 cm in diameter appears, firm and rarely painful [5]. The differential diagnosis generally considered is that of small tumors or hypodermic pseudotumors (lipoma, epidermoid cyst, etc.). Rare cases of nodule migration have been described [7].

Biology is of little help. General blood tests are normal. Due to the absence of blood dissemination, there is often no eosinophilia, no inflammatory syndrome, and no positive serology. Description of microfilariae in aspirated material or blood samples has been very rarely reported [3, 5, 8, 14, 18, 19].

Treatment is therefore primarily surgical removal, generally without antiparasitic systemic treatment. Some authors recommend supplementing surgical treatment by using antiparasitic treatment in the case of microfilariae in adult worms or recurrences, with no or limited evidence [1, 4, 5, 18, 20].

## 4. Anatomy and Histology

Formal diagnosis of the infection depends on the demonstration of the parasite in the resected specimen.

The nodule containing the parasite consists of a fibrous mass associated with moderate inflammatory changes (neutrophils, foreign body giant cells, lymphocytes), sometimes more intense in the case of suppurative necrosis [4, 20–22].

**Figure 2:**
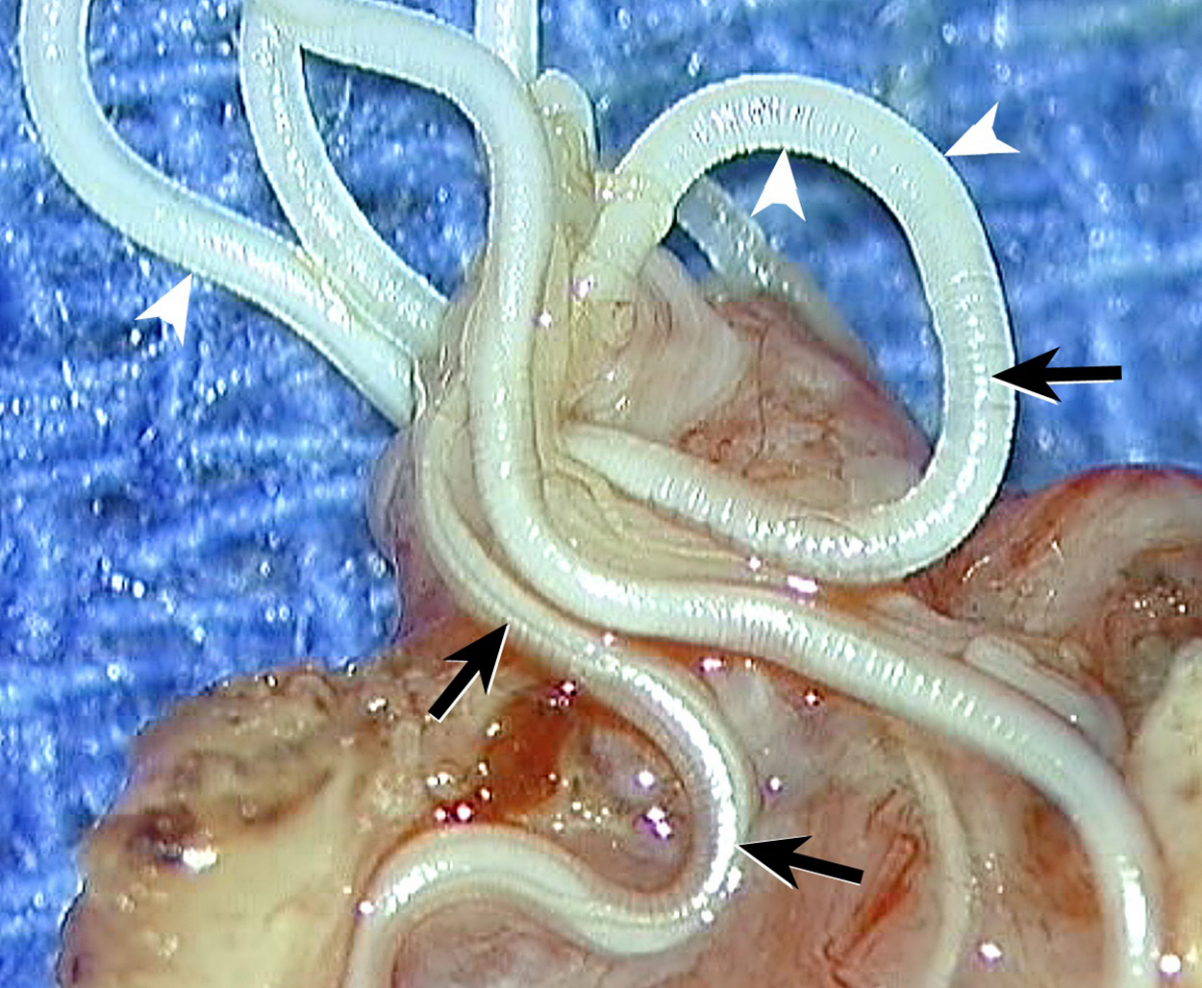
Picture of a *Dirofilaria repens* worm under operative microscope magnification. The image shows a surface with coarse longitudinal ridges (arrows) and numerous transverse annular strictures (arrowheads).

**Figure 3:**
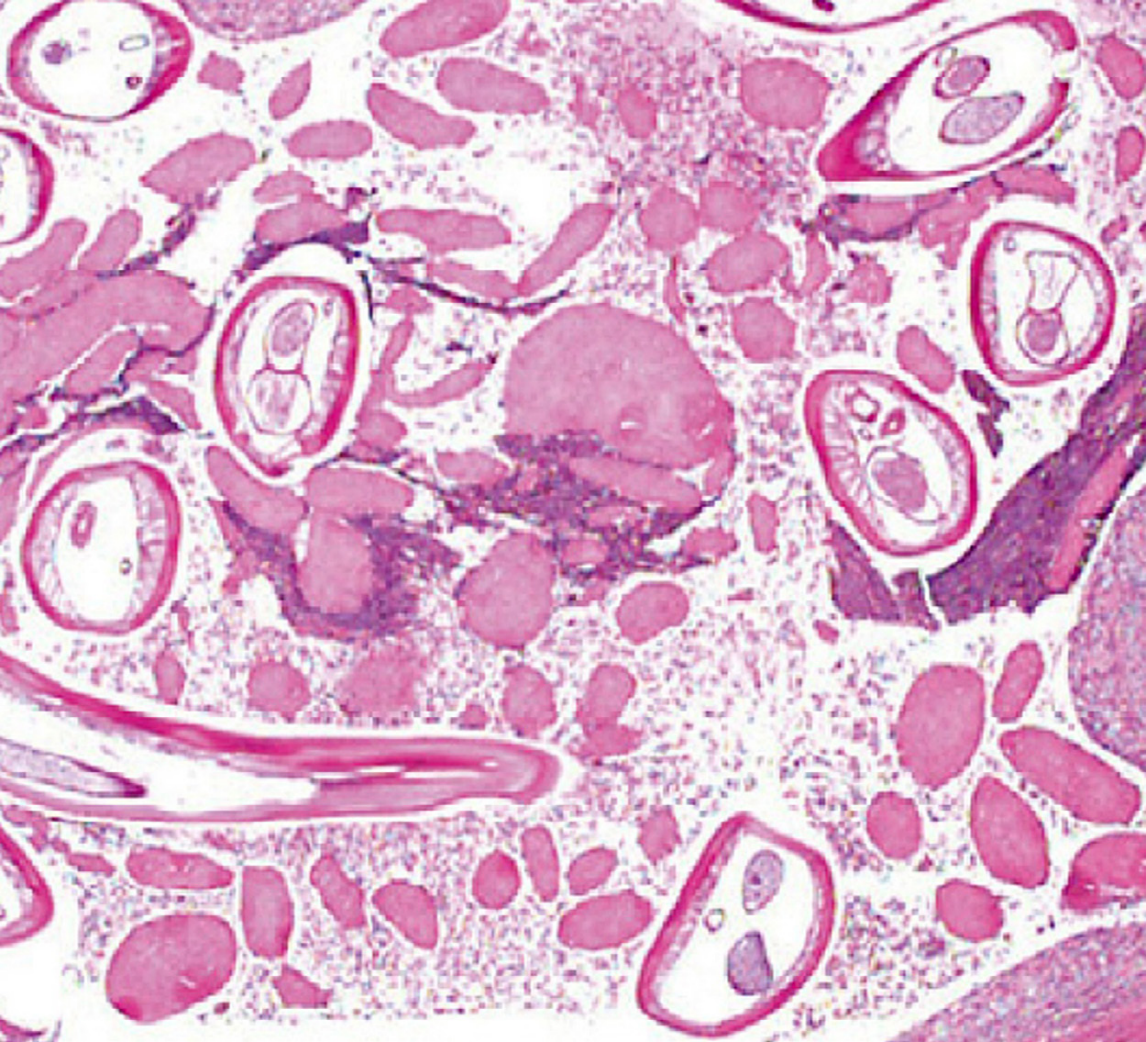
Histology of a *Dirofilaria repens*. Several sections of the worm show the peripheral cuticle covering a muscular layer. The central cavity contains the worm’s digestive tract and two genital tubes. The worm is located in a cystic cavity containing various inflammatory elements (Courtesy of C. Godfraind).

The identification of the species relies on the analysis of its diameter, peripheral musculature, and cuticle. *D. repens* is the largest of the species *Dirofilaria*. Its diameter (220–660 microns) is larger for the female. The length is around 10 cm. The cuticle has a surface adorned with transverse and longitudinal ridges (Figure 2), giving a scalloped appearance on cross-sections, unlike the completely smooth surface of *D. immitis*. Below are a muscular layer, a digestive tube, and two genital tubes (Figure 3) [1, 22]. If the filaria is dead, identification becomes difficult.

Nowadays, molecular biology can contribute to a very specific diagnosis with amplification and extraction by polymerase chain reaction (PCR), allowing in vitro production of numerous DNA fragments. Molecular techniques constitute a useful complementary tool, particularly when the morphology of the parasite is altered [5, 10, 18].

## 5. Imaging

Imaging of *D. repens* pseudotumors has been poorly described (34 publications in the PubMed research site in 2025).

On computed tomography (CT) with intravenous contrast, the lesion appears as a cystic formation surrounded by a wall of enhanced density [20, 23].

On magnetic resonance (MR), the lesion shows low signal intensity on T1-weighted images and high signal intensity on T2-weighted images. After intravenous administration of contrast material, the capsule and parts of the contents of the lesion show marked contrast enhancement, except for a central ill-defined structure which may represent the worm. These MR findings may suggest an inflammatory pseudotumor [21, 24].

At ultrasound, the appearance of the lesion can be more suggestive, identifying a hypoechoic mass containing double echogenic linear structures in addition to color Doppler signals [4, 8]. These double linear ‘rail’ images are explained by the visualization of the two reverberating walls of the parasite on either side of its central cavity. These double echogenic structures appear more or less long or short, or even point-like, depending on the orientation of the probe, which can be more or less longitudinal or transverse in relation to the parasite.

However, what is obviously the most specific sign at ultrasound is the visualization of the mobility of the content of the linear structures [4, 23, 25, 26]. Other authors did not mention this sign [22, 25]. The mobility of the echogenic structures is both a necessary and sufficient condition to formally conclude the presence of a parasite. However, this rail-like appearance is not entirely specific as it may be visible for other structures, which, as a distinctive feature, are immobile.

## 6. Clinical Case

A 42-year-old Italian man living in Belgium presents with a nodular swelling on the right temple, which had been almost asymptomatic for 3 years. His medical history includes trips to Egypt shortly before the appearance of the lesion. The swelling is firm, approximately 1 cm in diameter and 5 mm thick, with a volume that sometimes varies according to the patient.

**Figure 4:**
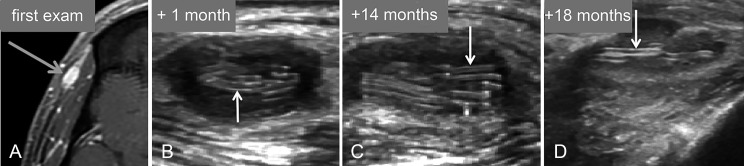
Temporal region examinations previously performed at other institutions. **(A)** T1-weighted MRI, performed after intravenous administration of contrast agent, shows a small hypodermal formation with a high signal corresponding to vascularized tissue (arrow). **(B)** to **(D)** Ultrasounds respectively performed 1 month, 14 months, and 18 months later all show the overall hypoechoic appearance of the lesions, containing curious fine echogenic structures in the shape of a ‘rail’ (arrows).

A previous MR scan had shown a small formation corresponding to vascularized tissue, and several previous ultrasounds had shown the presence of curious linear structures suggesting a foreign body (like surgical gauze) (Figure 4). However, this man has no history of local surgical procedures or other relevant medical history. He is in good general health.

**Figure 5:**
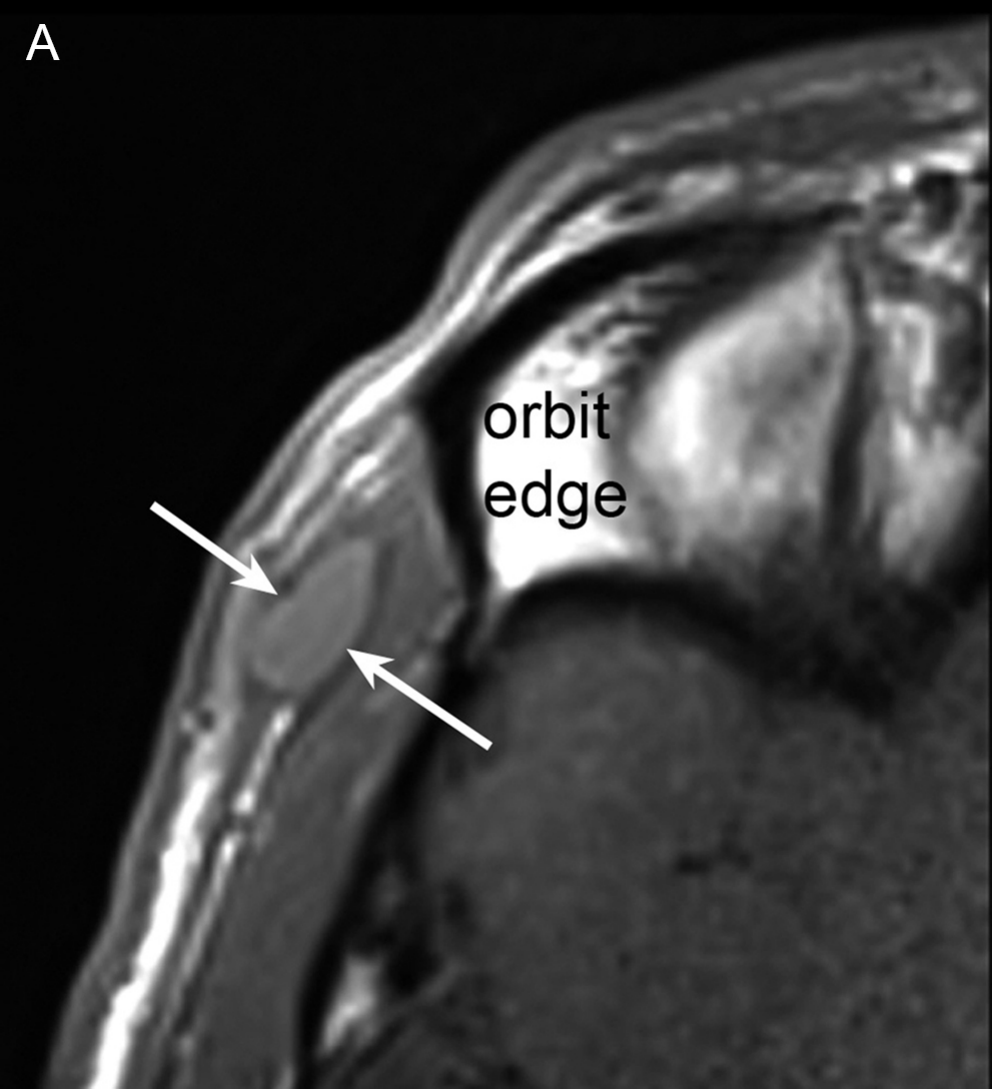

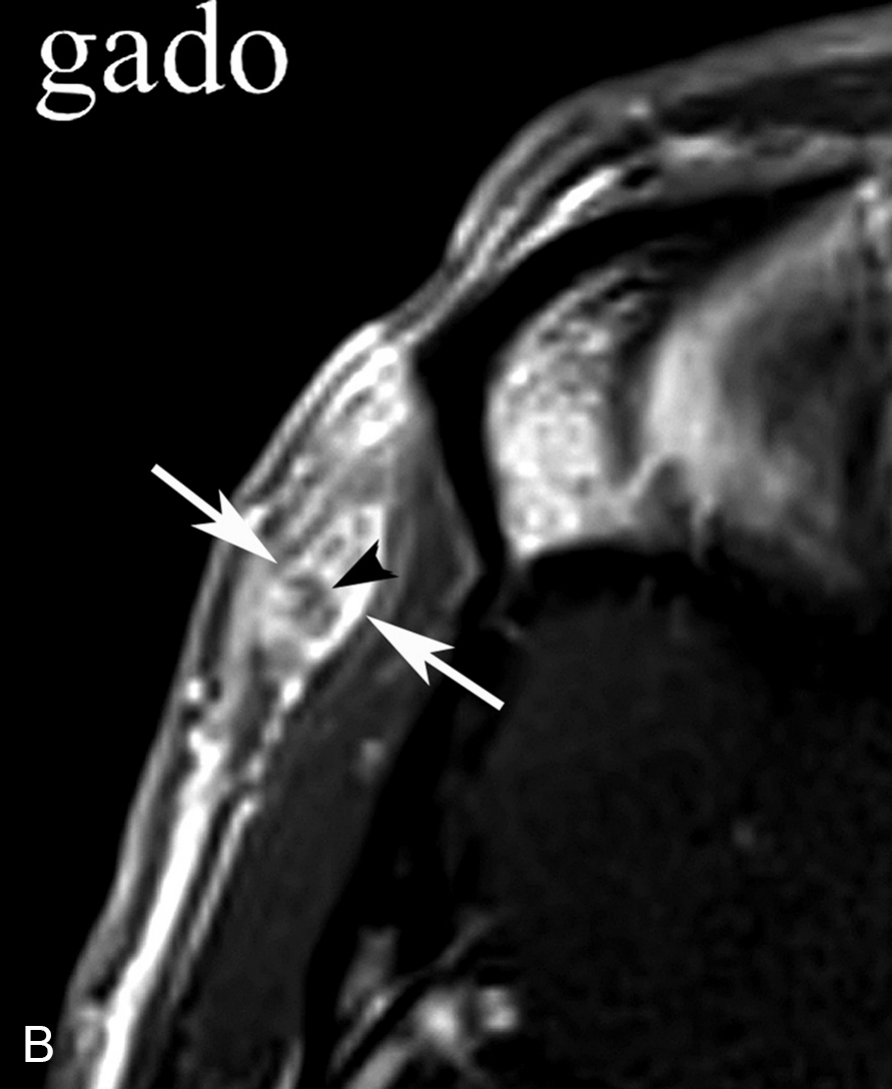

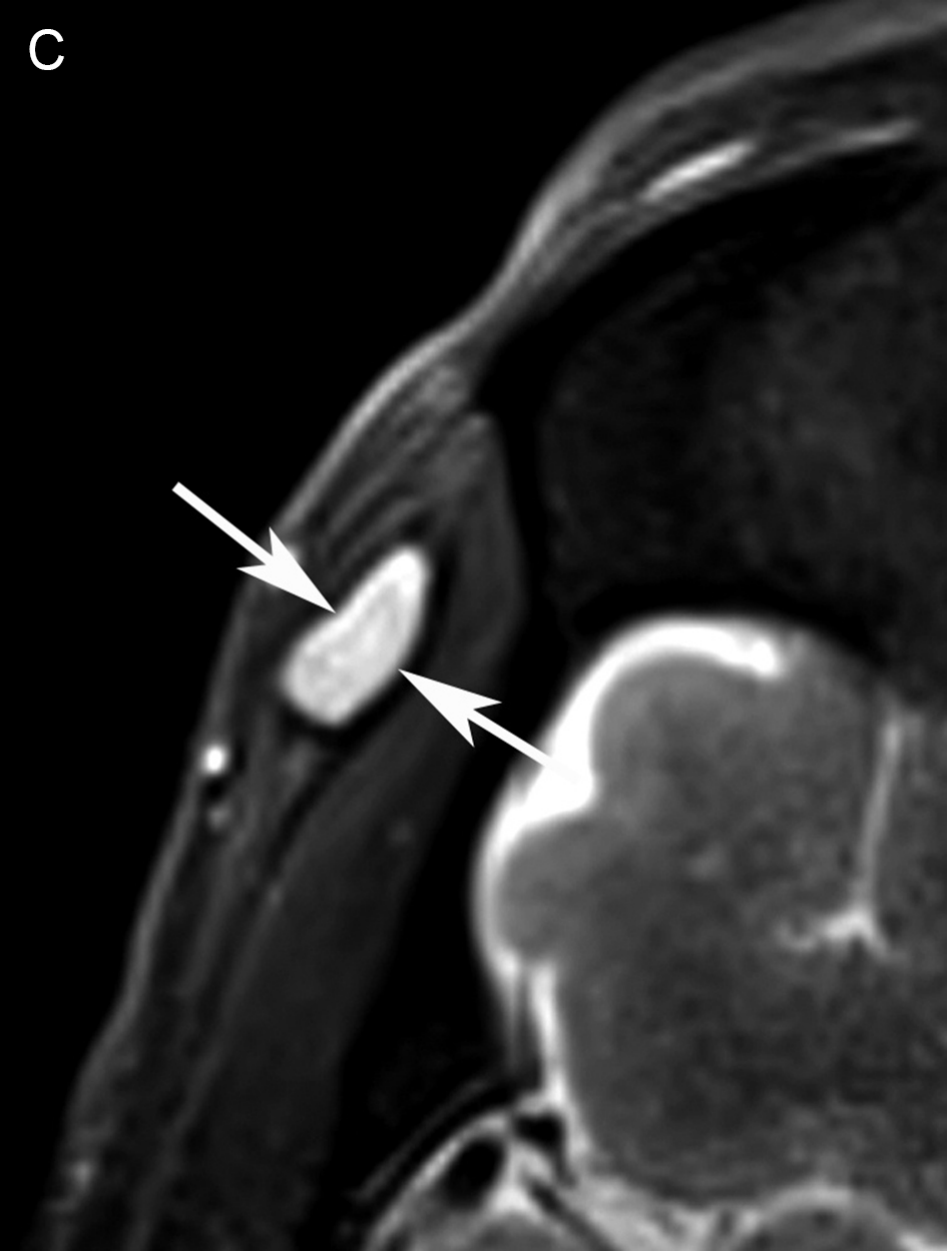
New MRI performed at our institution. The lenticular formation located in the deep hypodermal plane of the temporal region (arrows) shows a low to intermediate signal on T1-weighted image **(A)**, with signal enhancement after contrast injection **(B)**, and a high signal on T2-weighted images with fat saturation **(C)**. Note the heterogeneity of the central portion of the mass, which contains small unenhanced areas (black arrowhead in **B**).

**Figure 6:**
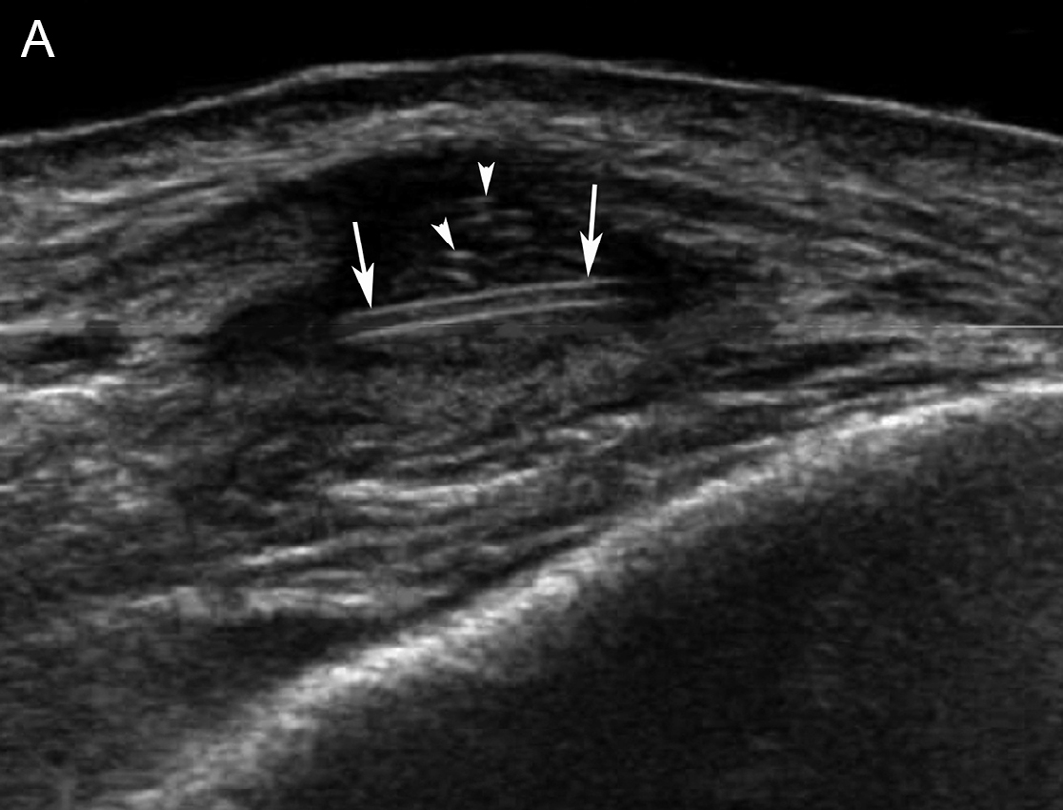

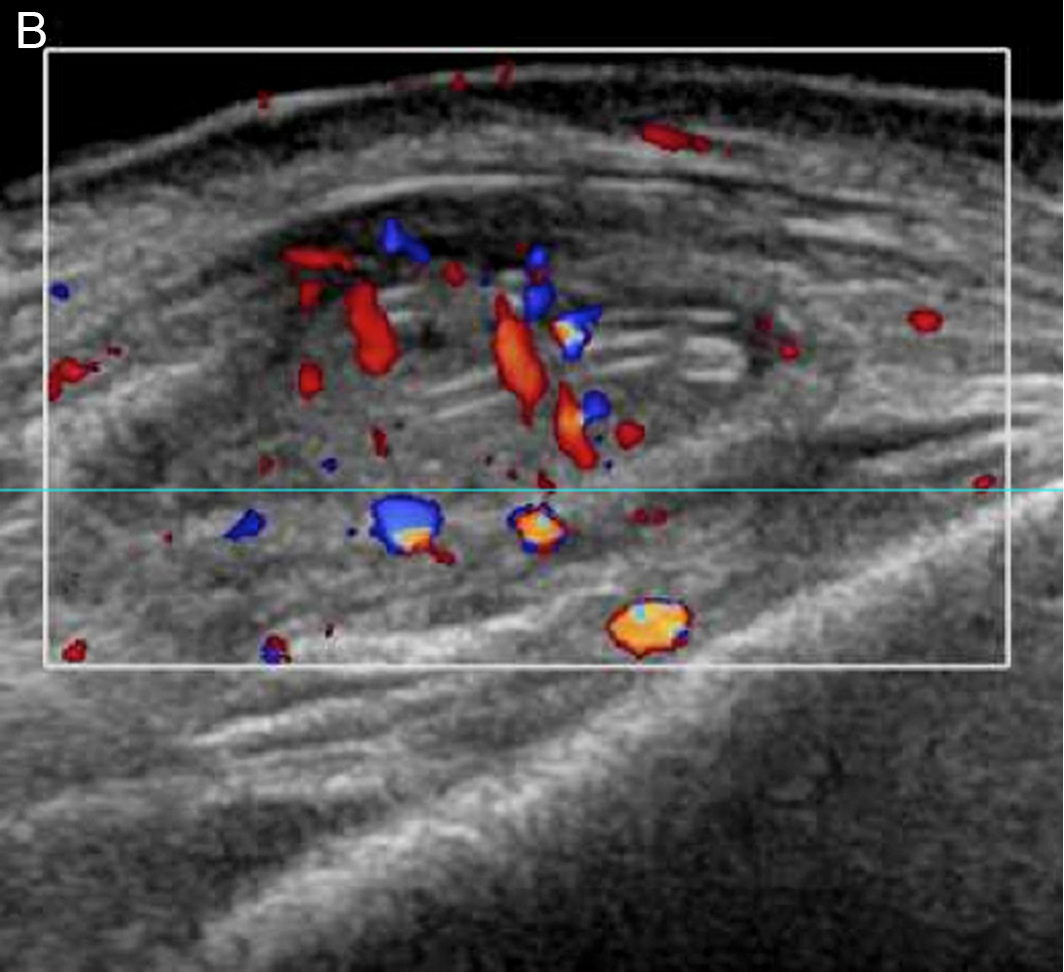

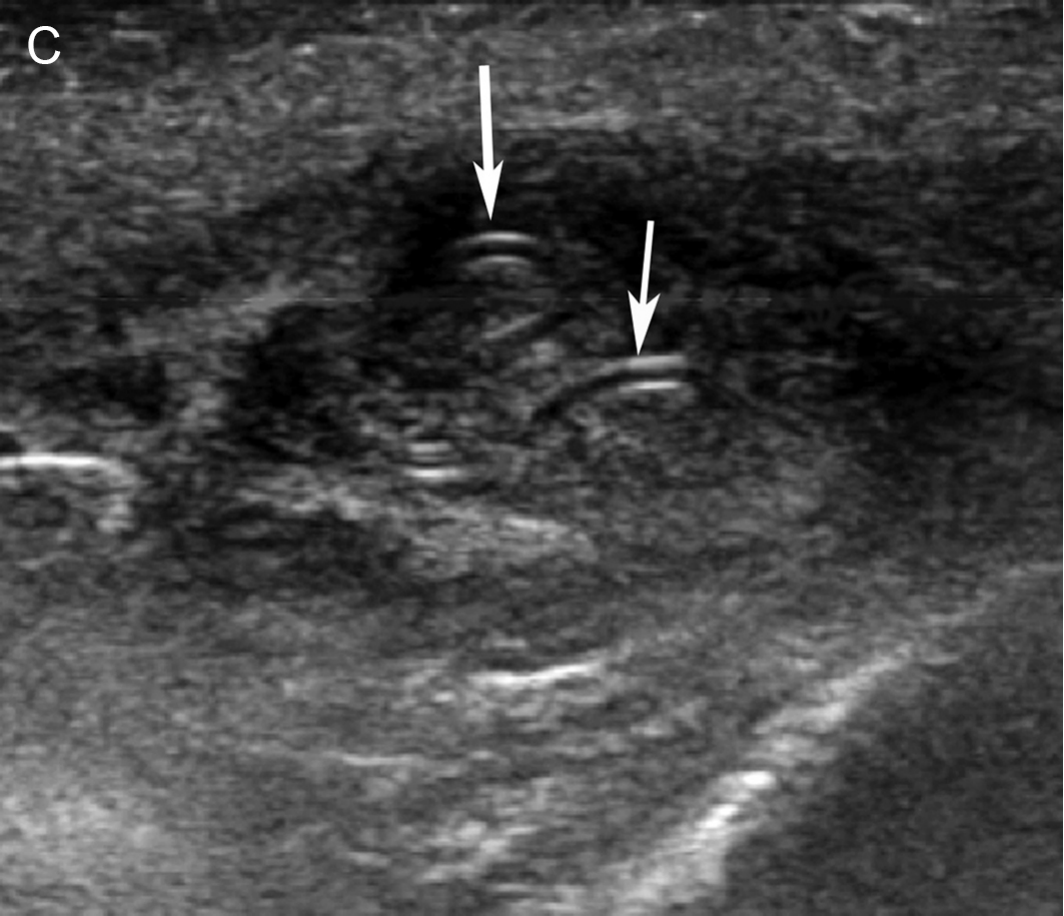
A new ultrasound scan performed immediately after the new MRI. **(A)** Conventional images perpendicular to the skin surface show a poorly defined, heterogeneous, hypoechoic deep hypodermal mass containing rectilinear double echogenic structures several millimeters long (arrows) or very short, almost point-like (arrowheads). **(B)** Color Doppler examination shows clear hypervascularization. **(C)** A nearly ‘horizontal’ slice, close to a plane parallel to the skin, taken after more than 10 minutes of examination, shows that the double echogenic structure has curved portions (arrows). It is at this stage that spontaneous movements of the structure were observed (see Figure 7).

There was no inflammation at blood test and serology was negative for various parasites. An antihelminthic treatment has already been prescribed, without results. After discussion, excision is decided and a new imaging examination is performed before surgery. MRI (Figure 5) shows a small deep hypodermic mass, with low signal on T1, relatively high on T2, and marked signal enhancement on T1 with gadolinium. Repeated ultrasound (Figure 6) shows a moderately hypoechoic lesion, with color Doppler signals and numerous linear echogenic structures with a double line appearance, approximately 0.6 mm thick. The length of these ‘rail-like’ structures appears very variable. They are either elongated or nearly punctiform, suggesting tubular structures viewed more or less longitudinally or transversely.

**Figure 7:**
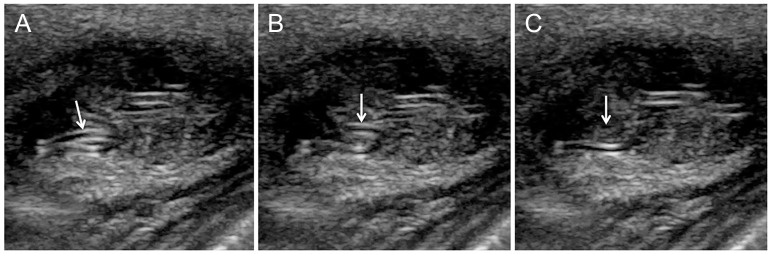
Images extracted from a short ultrasound video taken late with the motionless probe almost tangent to the skin plane, show that certain echogenic structures move and then disappear (arrowheads in **A**, **B**, and **C**), resulting from their spontaneous mobility.

Sections made in a nearly ‘horizontal’ plane (close to a plane parallel to the skin) show that these linear structures have curved segments (Figure 6c). During the ultrasound imaging process, more than 10 minutes after the start of the examination, the tubular structures exhibited spontaneous undulating movements, very suggestive of a living parasite (Figure 7).

**Figure 8:**
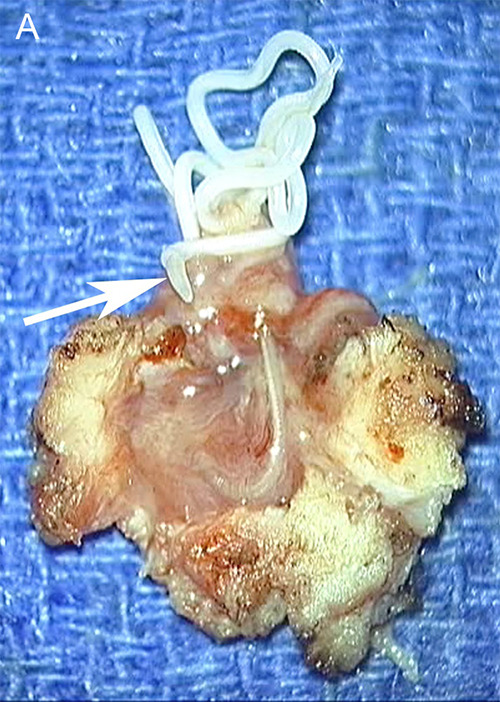

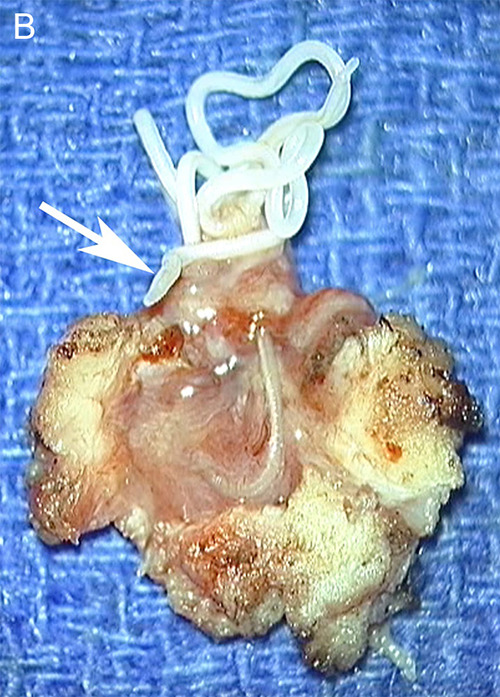

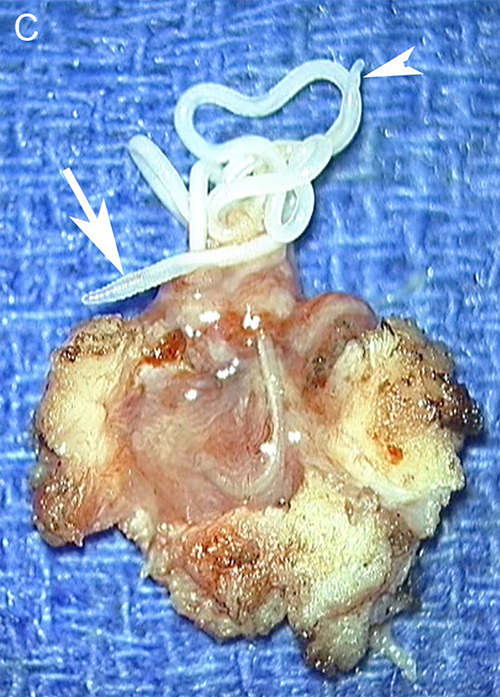

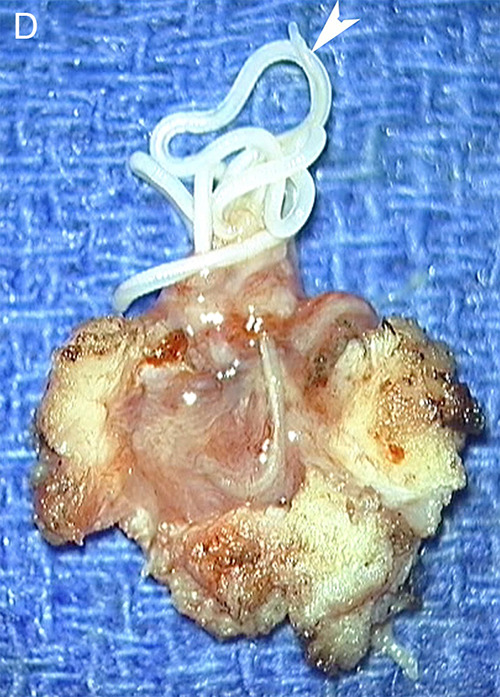
Photographs taken from a video of the open surgical excision specimen. A whitish, threadlike structure corresponding to the worm emerges from the tissue mass through a breach. Some portions of the worm are mobile (arrows in **A**, **B**, and **C** and arrowheads in **C** and **D**).

The surgical excision of the nodule revealed the presence of a long whitish worm (Figure 8), spontaneously mobile, measuring 12 cm in length, corresponding to descriptions of *D. repens*. The subsequent evolution was very favorable.

It should be noted that patience may be essential. In our observation, the spontaneous movements of these linear structures were only observed after more than 10 minutes of examination. But it is obviously possible that, in other cases, parasite mobility is observed earlier, or even later.

The mobility of the echogenic structures is both a necessary and sufficient condition to formally conclude the presence of a parasite. Indeed, other ‘rail’ images can be produced by other structures, but they are immobile.

It might be added that the curved appearance of the parasite’s structure in sections taken in a plane almost parallel to the skin could be an additional sign indicating the flexibility of the worm’s structure if not compressed by the pressure of the skin surface.

## 7. Conclusions

*D. repens* pseudotumors are relatively rare and their pathology is still poorly known, probably due to the insidious nature of the infection.

The number of cases has increased considerably in recent decades, and their geographical distribution, once concentrated in southern Europe, is currently spreading northward and eastward. This increase is due to several factors, including global warming.

Diagnosis by imaging is based on the detection of a small, nonspecific tissue mass on CT and MRI scans, and especially on ultrasound visualization of double linear ‘rail’ images corresponding to the worm’s walls. This aspect is not specific, but can become so if one is fortunate enough to detect its mobility that is the most conclusive diagnostic element. In our observation, these spontaneous movements were only observed after more than 10 minutes of examination.
